# Supplementary material for: Contribution of epigenetic landscapes and transcription factors to X-chromosome reactivation in the inner cell mass
Source: Nat Commun. 2017 Nov 3;8:1297. doi: 10.1038/s41467-017-01415-5 (PMC5670228; doi:10.1038/s41467-017-01415-5)
Supplement: Supplementary file 1 — Supplementary Information [file 41467_2017_1415_MOESM1_ESM.pdf]

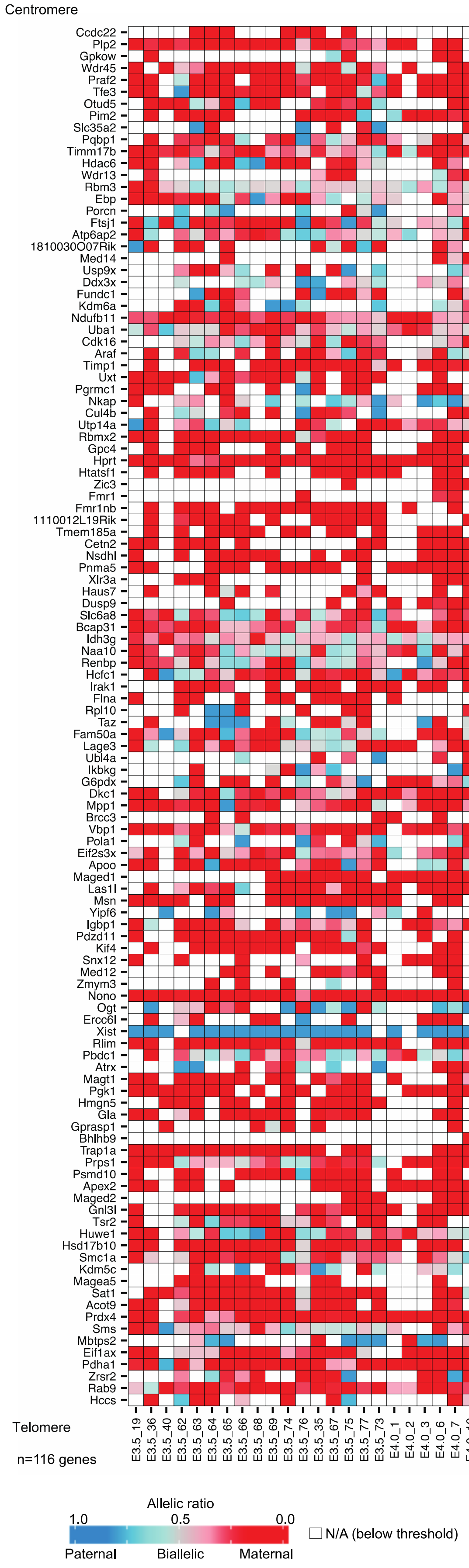
Centromere
Ccdc22
Plp2
Gpkow
Wdr45
Praf2
Tfe3
Otud5
Pim2
Slc35a2
Pqbp1
Timm17b
Hdac6
Wdr13
Rbm3
Ebp
Porcn
Ftsj1
Atp6ap2
1810030O07Rik
Med14
Usp9x
Ddx3x
Fundc1
Kdm6a
Ndufb11
Uba1
Cdk16
Araf
Timp1
Uxt
Pgrmc1
Nkap
Cul4b
Utp14a
Rbmx2
Gpc4
Hprt
Htatsf1
Zic3
Fmr1
Fmr1nb
1110012L19Rik
Tmem185a
Cetn2
Nsdhl
Pnma5
Xlr3a
Haus7
Dusp9
Slc6a8
Bcap31
Idh3g
Naa10
Renbp
Hcfc1
Irak1
Flna
Rpl10
Taz
Fam50a
Lage3
Ubl4a
Ikbkg
G6pdx
Dkc1
Mpp1
Brcc3
Vbp1
Pola1
Eif2s3x
Apoo
Maged1
Las1l
Msn
Yipf6
Igbp1
Pdzd11
Kif4
Snx12
Med12
Zmym3
Nono
Ogt
Ercc6l
Xist
Rlim
Pbdc1
Atrx
Magt1
Pgk1
Hmgn5
Gla
Gprasp1
Bhlhb9
Trap1a
Prps1
Psmd10
Apex2
Maged2
Gnl3l
Tsr2
Huwe1
Hsd17b10
Smc1a
Kdm5c
Magea5
Sat1
Acot9
Prdx4
Sms
Mbtps2
Eif1ax
Pdha1
Zrsr2
Rab9
Hccs
Telomere
n=116 genes
E3.5_19
E3.5_36
E3.5_40
E3.5_62
E3.5_63
E3.5_64
E3.5_65
E3.5_66
E3.5_68
E3.5_69
E3.5_74
E3.5_76
E3.5_35
E3.5_67
E3.5_75
E3.5_77
E3.5_73
E4.0_1
E4.0_2
E4.0_3
E4.0_6
E4.0_7
E4.0_10
E4.0_11
E4.0_57
E4.0_59
E4.0_63
E4.0_85
E4.0_86
E4.0_87
E4.0_5
E4.0_8
E4.0_9
E4.0_12
E4.0_13
E4.0_14
E4.0_15
E4.0_16
E4.0_17
E4.0_90
Allelic ratio
1.0
0.5
0.0
Paternal
Biallelic
Maternal
N/A (below threshold)

Supplementary Figure 1 (related to Figure 4)

Heatmap representing the allele-specific expression of informative and well expressed X-linked genes in each single cell, in E3.5 (Trophectoderm and ICM) and E4.0 (Primitive Endoderm and Epiblast) female hybrid embryos (B6 x Castaneus cross). Strictly maternally expressed genes (allelic ratio ≤0.15) are represented in red and strictly paternally expressed genes (allelic ratio ≥0.85) in blue. Colour gradients are used in between and genes have been ordered by genomic position. n=116 genes.

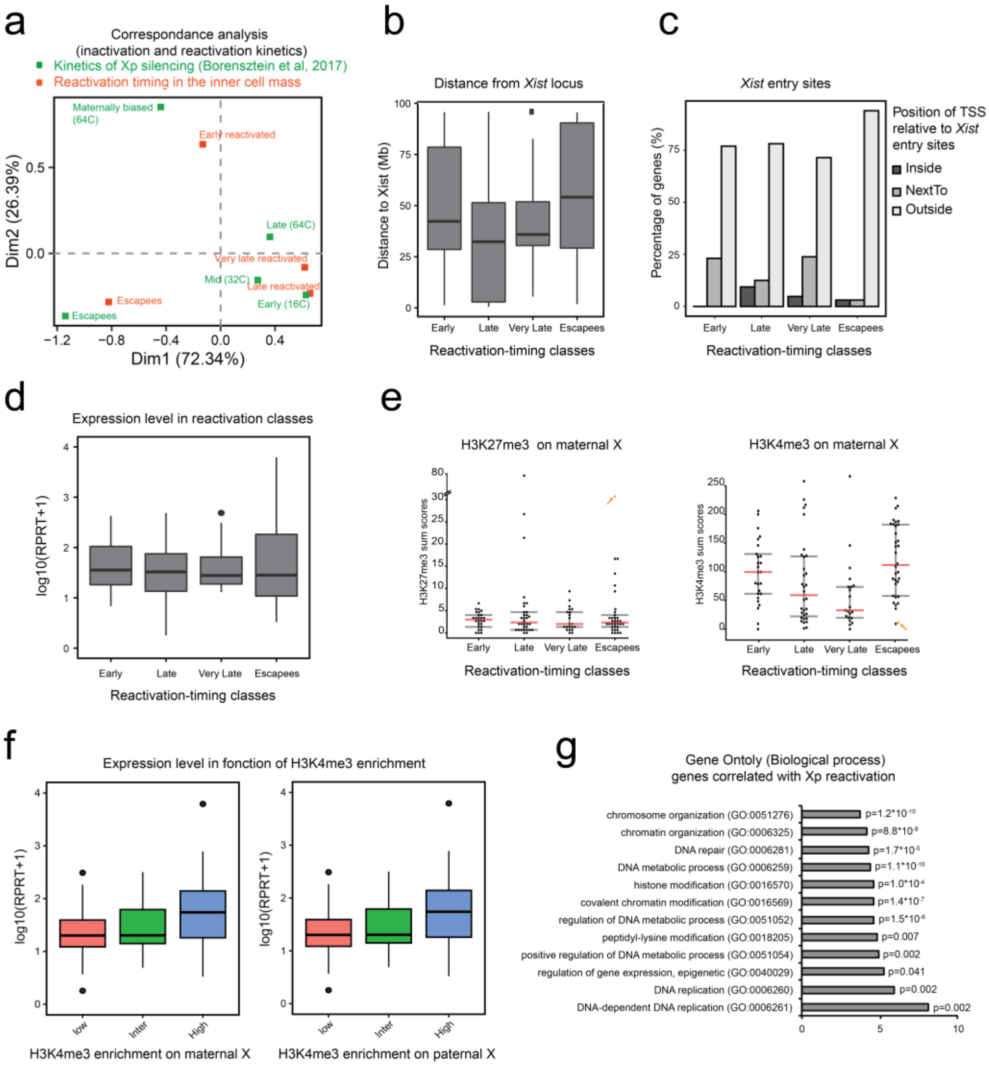
a
Correspondance analysis
(inactivation and reactivation kinetics)
Kinetics of Xp silencing (Borensztein et al, 2017)
Reactivation timing in the inner cell mass
Maternally biased (64C)
Early reactivated
Late (64C)
Very late reactivated
Mid (32C)
Late reactivated
Early (16C)
Escapees
Escapees
Dim2 (26.39%)
Dim1 (72.34%)
b
Distance from Xist locus
Distance to Xist (Mb)
Early
Late
Very Late
Escapees
Reactivation-timing classes
c
Xist entry sites
Percentage of genes (%)
Position of TSS relative to Xist entry sites
Inside
NextTo
Outside
Early
Late
Very Late
Escapees
Reactivation-timing classes
d
Expression level in reactivation classes
log10(RPRT+1)
Early
Late
Very Late
Escapees
Reactivation-timing classes
e
H3K27me3 on maternal X
H3K27me3 sum scores
Early
Late
Very Late
Escapees
Reactivation-timing classes
H3K4me3 on maternal X
H3K4me3 sum scores
Early
Late
Very Late
Escapees
Reactivation-timing classes
f
Expression level in fonction of H3K4me3 enrichment
log10(RPRT+1)
low
Inter
High
H3K4me3 enrichment on maternal X
log10(RPRT+1)
low
Inter
High
H3K4me3 enrichment on paternal X
g
Gene Ontoly (Biological process)
genes correlated with Xp reactivation
chromosome organization (GO:0051276) p=1.2*10-10
chromatin organization (GO:0006325) p=8.8*10-9
DNA repair (GO:0006281) p=1.7*10-5
DNA metabolic process (GO:0006259) p=1.1*10-10
histone modification (GO:0016570) p=1.0*10-4
covalent chromatin modification (GO:0016569) p=1.4*10-7
regulation of DNA metabolic process (GO:0051052) p=1.5*10-6
peptidyl-lysine modification (GO:0018205) p=0.007
positive regulation of DNA metabolic process (GO:0051054) p=0.002
regulation of gene expression, epigenetic (GO:0040029) p=0.041
DNA replication (GO:0006260) p=0.002
DNA-dependent DNA replication (GO:0006261) p=0.002

Supplementary Figure 2 (related to Figure 4)

(a) Correspondence analysis (CA) of X-linked gene reactivation and silencing classes based on their timing of reactivation in ICM and timing of silencing during imprinted XCI in pre-implantation embryos as previously determined in Borensztein *et al,* 2017[7].

(b) Distance to *Xist* genomic locus. Distribution of the genomic distances to *Xist* locus (in Mb) for the different X-linked gene reactivation classes. Transcription Start Site (TSS) of each gene has been used to measure the distance to *Xist* locus. Non-significant by Kruskal-Wallis test. Boxplot represent median with lower and upper quartiles.

(c) Percentage of X-linked genes from the different reactivation classes classified by their relative position to *Xist* "entry" sites (as identified during XCI induction in ESCs[37]: "inside" (TSS located in a *Xist* "entry" site), "next to" (TSS located less than 100 kb to an "entry" site) and "outside" (over 100 kb from an "entry" site). Non-significant by Kruskal-Wallis test.

(d) Expression level of X-linked genes in the different reactivation-timing classes in E3.5 ICM samples (mean of each single gene). No differences in expression level can be seen for early reactivated and escapees genes compared to late and very late genes. Boxplot represent median with lower and upper quartiles. Non-significant by Kruskal-Wallis test.

(e) Enrichment of H3K27me3 and H4K4me3 on maternal X obtained from (Zheng et al., 2016)[38]. Each dot represents a single gene. *Xist* dot is highlighted with an orange arrow. No differences can be seen for H3K27me3 distribution in any reactivation-timing groups (by Wilcoxon test), contrary to the paternal X (Figure 5b). Enrichment of H3K4me3 is much higher on maternal X chromosome compared to all paternal X (Figure 5b). Very late genes are significantly different compared to Early and Escapee groups for H3K4me3 maternal enrichment by Wilcoxon test (respectively $p=3.61*10^{-3}$ and $p=1.71*10^{-4}$).

(f) Expression level of X-linked genes in function of their enrichment of H3K4me3 on

maternal (left) and paternal (right) X chromosomes. Low, intermediate (Inter) and highly (High) enriched classes have been designed by H3K4me3 sum scores <5, 5≤ and ≥ 15, and >15 respectively. On the maternal and paternal X chromosomes, lowly enriched genes for H3K4me3 marks are significantly less expressed than highly expressed ones (respectively p=0.028 and p=0.045, by Dunn's test). Boxplot represent median with lower and upper quartiles.

(g) Representation of the Gene ontology analysis of Biological process performed on the best correlated genes with X-linked gene reactivation (q-value <0.05, Supplemental Table 3). The twelve best enrichment classes (based on fold enrichment) are represented with their p-value.

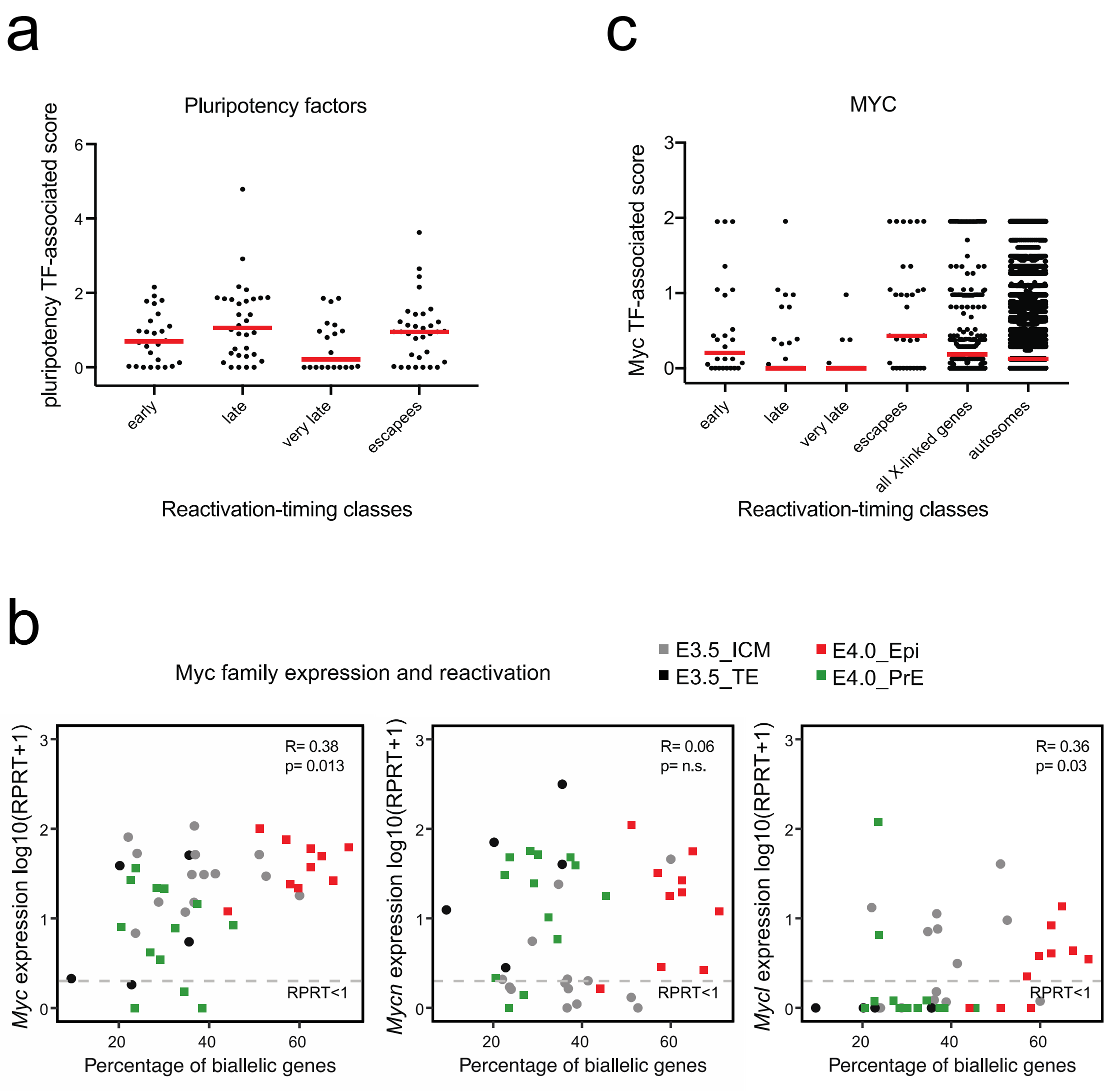
a
Pluripotency factors
pluripotency TF-associated score
early
late
very late
escapees
Reactivation-timing classes
c
MYC
Myc TF-associated score
early
late
very late
escapees
all X-linked genes
autosomes
Reactivation-timing classes
b
Myc family expression and reactivation
E3.5_ICM
E3.5_TE
E4.0_Epi
E4.0_PrE
Myc expression log10(RPRT+1)
R= 0.38
p= 0.013
RPRT<1
Percentage of biallelic genes
Mycn expression log10(RPRT+1)
R= 0.06
p= n.s.
RPRT<1
Percentage of biallelic genes
Mycl expression log10(RPRT+1)
R= 0.36
p= 0.03
RPRT<1
Percentage of biallelic genes

Supplementary Figure 3 (related to Figure 4)

(a) The sum of TF-gene associated score of pluripotency factors (Nanog, Esrrb, Klf4, Oct4, Sox2, Tcfcp211) per gene and obtained from Chen et al., 2008[40], is presented in each reactivation-timing groups (median in red). There is no significant difference between the groups according to Kruskal-Wallis test.

(b) The level of expression of *Myc* genes (*Myc, Mycn* and *Mycl*) is plotted in function of the number of biallelically/reactivated X-linked genes in each single cell. Different colours are applied for E3.5 trophectoderm (TE), E3.5 ICM, E4.0 Primitive endoderm (PrE) and E4.0 Epiblast (Epi) cells. By Spearman correlation, a positive correlation is seen between level of expression of *Myc* and *Mycl* and high percentage of biallelically expressed genes. Genes with level of expression as (RPRT<1) are considered as non-expressed in our samples.

(c) The sum of TF-gene associated score of Myc factors (Myc and Mycn) per gene and obtained from Chen et al., 2008[40], is presented in each reactivation-timing groups, for all the X-linked genes and for all the autosomal genes (median in red). Late and very late reactivated genes are significantly depleted in Myc-associated score compared to the other classes, $p<0.0001$ by Kruskal-Wallis test, and are then potentially less responsive to Myc transcription factors than early-reactivated genes and escapees.

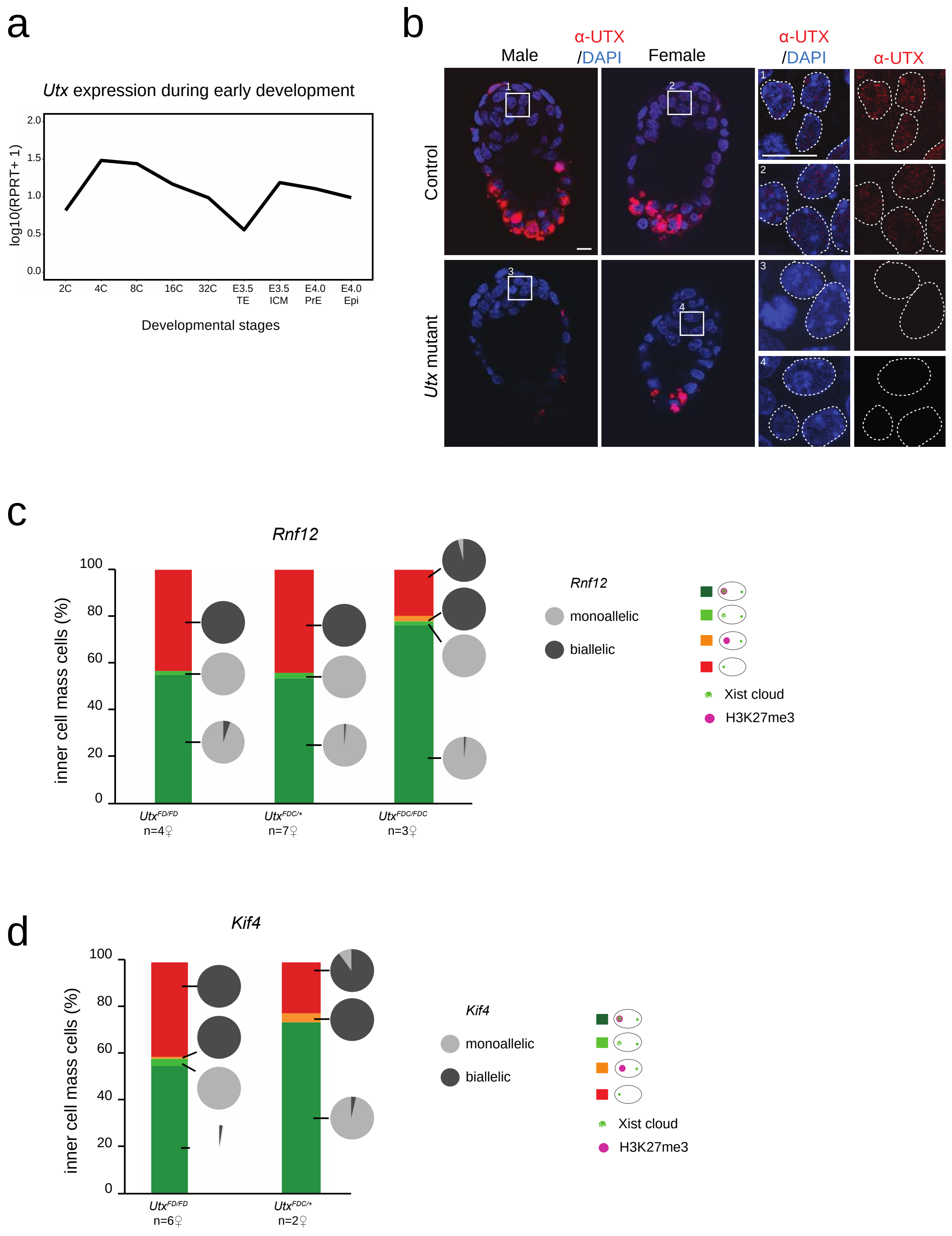
a
Utx expression during early development
log10(RPRT+ 1)
2.0
1.5
1.0
0.5
0.0
2C
4C
8C
16C
32C
E3.5 TE
E3.5 ICM
E4.0 PrE
E4.0 Epi
Developmental stages
b
Male
α-UTX /DAPI
Female
α-UTX /DAPI
α-UTX
Control
Utx mutant
1
2
3
4
c
Rnf12
inner cell mass cells (%)
100
80
60
40
20
0
UtxFD/FD n=4♀
UtxFDC/+ n=7♀
UtxFDC/FDC n=3♀
Rnf12
monoallelic
biallelic
Xist cloud
H3K27me3
d
Kif4
inner cell mass cells (%)
100
80
60
40
20
0
UtxFD/FD n=6♀
UtxFDC/+ n=2♀
Kif4
monoallelic
biallelic
Xist cloud
H3K27me3

Supplementary Figure 4 (related to Figure 5)

(a) Level of expression of *Utx* gene during preimplantation mouse development (expression mean of all single cells). *Utx* is downregulated in trophectoderm but stay expressed in ICM cells at E3.5.

(b) Maximum intensity projection of 1.5 µm section for control *(*$Utx^{FD/FD}$ female and $Utx^{FD/Y}$ male) and mutant ($Utx^{FDC/FDC}$ female and $Utx^{FDC/Y}$ male) E4.5 blastocysts analysed by immunofluorescence against UTX (red). DAPI is in dark blue. Enlarged nuclei are shown. Scale bars represent 10µm.

(c) Proportion (mean) of ICM cells showing enrichment of H3K27me3 on the Xist RNA coated X chromosome from E4.5 control *(*$Utx^{FD/FD}$), heterozygous *(*$Utx^{FDC/+}$*)* and mutant ($Utx^{FDC/FDC}$) female blastocysts, linked with *Rnf12* allelic status.

(d) Proportion (mean) of ICM cells showing enrichment of H3K27me3 on the Xist RNA coated X chromosome) from E4.5 control *(*$Utx^{FD/FD}$) and mutant ($Utx^{FDC/FDC}$) female blastocysts, linked with *Kif4* allelic status.

Antibodies for Immunofluorescent analysis.

Primary antibody	Dilution ratio	Company	Cat. No.	Species Reactivity in Product Data-sheet
Goat polyclonal anti-Human GATA6	1/200	R&D	AF1700	Human
Rat monoclonal anti-Mouse Nanog	1/200	eBioscience	14-5761-80	Mouse
Mouse moclonal anti-Human UTX	1/200	Abcam	ab184525	Human
Mouse moclonal anti-H3K27me3	1/100	Gift from D. Reinberg	7B11	Mouse
Alexa 488 Donkey anti-mouse IgG	1/500	Life Technologies	A212020	Mouse
Alexa 647 Donkey anti-mouse IgG	1/500	Life Technologies	A31571	Mouse
Alexa 568 Donkey anti-goat IgG	1/500	Life Technologies	A11057	Goat
Alexa 488 Donkey anti-rat IgG	1/500	Life Technologies	A21208	Rat

BAC Clone for RNA FISH probes

Gene	Clone ID
Abcb7	RP24-274B9
Atp6ap2	RP23-43D10
Atp7a	RP23-186F4
Atrx	RP23-265D6
Atrx	WI1-2039P10
Fmr1	Rp24-183G11
Kif4	RP23-248G16
Pdha1	RP24-211E22
Rnf12	RP24-240J16

Supplementary Table 1: List of RNA-FISH probes and antibodies
