# Supplementary material for: Contribution of epigenetic landscapes and transcription factors to X-chromosome reactivation in the inner cell mass
Source: Nat Commun. 2017 Nov 3;8:1297. doi: 10.1038/s41467-017-01415-5 (PMC5670228; doi:10.1038/s41467-017-01415-5)
Supplement: Supplementary file 3 — Description of Additional Supplementary Files [file 41467_2017_1415_MOESM3_ESM.pdf]

Description of Additional Supplementary Files

File Name: Supplementary Data 1
Description: Summary of single cell RNAseq samples. For each library is provided: single cell's name, stage, embryo number, gender, cross and the raw read number, filtered ones and percentage of mapping.

File Name: Supplementary Data 2
Description: Silencing gene classes Reactivation timing and allelic ratio for the 116 informative and well-expressed Xlinked genes in hybrid ICM cells (B6 x Cast cross).

File Name: Supplementary Data 3
Description: List of genes correlated or anti-correlated with X-linked gene reactivation between E3.5 and E4.0.
